# Are *Cristaria herculea* (Middendorff, 1847) and *Cristaria plicata* (Leach, 1815) (Bivalvia, Unionidae) separate species?

**DOI:** 10.3897/zookeys.438.7493

**Published:** 2014-09-01

**Authors:** Olga K. Klishko, Manuel Lopes-Lima, Elsa Froufe, Arthur E. Bogan

**Affiliations:** 1 Institute of Natural Resources, Ecology and Criology, Russian Academy of Sciences Siberian Branch, Chita 672014, Russia; 2 Interdisciplinary Centre of Marine and Environmental Research (CIIMAR/CIMAR), University of Porto, Rua dos Bragas 289, 4050-123 Porto, Portugal; 3 Institute of Biomedical Sciences Abel Salazar (ICBAS), University of Porto, Rua de Jorge Viterbo Ferreira 228, 4050-313 Porto, Portugal; 4 Mollusc Specialist Group, Species Survival Commission, International Union for Conservation of Nature (SSC/IUCN), c/o IUCN, 219 Huntingdon Road, Cambridge, United Kingdom; 5 Research Laboratory, North Carolina State Museum of Natural Sciences, MSC 1626, Raleigh, NC 27699-1626, United States of America

**Keywords:** Bivalvia, Unionidae, Anodontini, CO1, Transbaikalia, Russia

## Abstract

The number of species in the freshwater mussel genus *Cristaria* Schumacher, 1817 recognized from Far East Russia has varied over the last several decades. While some authors consider the occurrence of only one species, *Cristaria plicata* (Leach, 1815), widespread in East Asia, others, recognize two separate species *Cristaria herculea* (Middendorff, 1847) and *Cristaria tuberculata* Schumacher, 1817 from Far East Russia, distinct from *C. plicata*. ﻿For the present study, freshwater mussels, identified as *C. herculea*,﻿ were collected in the Upper Amur basin (Transbaikalia, Russia). The shell morphology and the whole soft body anatomy were analysed in detail and compared with previously published information on other *Cristaria* spp.. Additionally, a cytochrome oxidase subunit 1 (CO1) gene fragment was sequenced from foot tissue samples of selected animals, collected from the same region, and compared with published data. Based upon morphological similarities of glochidia and adult morphology and anatomy as well as the mitochondrial DNA sequence analysis, we consider *C. herculea* as a synonym of *C. plicata*. ﻿Further analysis of Far East Russia *C. herculea* and *C. tuberculata* specimens using both molecular and morphological characters should be carried in the future to enhance our knowledge about the taxonomy within the *Cristaria* genus. Moreover, a comprehensive revision of the genus *Cristaria* is needed, restricting the type locality and comparing topotypic specimens for both *C. plicata* and *C. tuberculata*, and including all recognized *Cristaria* species.

## Introduction

Freshwater bivalves of the Unionidae provide important ecosystem functions and services (Vaughn and Hakenkamp 2001; Aldridge et al. 2007). However, many of their populations are in decline and this faunistic group is presently among the most threatened worldwide (Bogan 1993). In terms of conservation, it is essential to have a classification system that reflects the freshwater bivalve taxonomic diversity as well as their evolutionary relationships. Over the last decade, there have been an increasing number of taxonomy papers reflecting phylogenetic patterns with the aid of molecular tools. However, most included North American and European taxa. On the other hand, other taxa e.g. from the Southern Hemisphere or East Asian countries have been neglected and are still poorly studied. This is the case of *Cristaria* Schumacher, 1817, a relatively widespread genus in South East Asia, where its interspecific and intraspecific phylogenetic relationships are still not well understood.

The taxonomy and status of *Cristaria* species in Far East Russia has not been consistent among malacologists. While some authors, consider the presence of only one species, *Cristaria plicata* (Leach, 1815) which is widespread in Eastern Asia, from Russia (Amur River basin and Khanka Lake) to Japan and south to South Korea, China, Vietnam, Lao People’s Republic, Thailand and Cambodia (Zhadin 1938, 1965; Haas 1969; Brandt 1974; Đặng et al. 1980; Kondo 2008; He and Zhuang 2013), others, consider *Cristaria herculea*, *Cristaria tuberculata*,﻿ and *Cristaria plicata* are separate species (Sayenko et al. 2005). *Cristaria herculea*,﻿ with a laterally compressed shell, is widespread in the whole Amur River basin including the Zeya, the Argun, the Nercha, the Shilka and the Onon rivers as well as Khanka Lake and Buir-Nur Lake (Mongolia) (Zatravkin and Bogatov 1987; Starobogatov et al. 2004; Klishko 2012). The other species recognized in Far East Russia, with an inflated shell, *Cristaria tuberculata* Schumacher, 1817, is limited to the Far East Russia in Khanka Lake and the Ussury River basin (Moskvicheva 1973; Zatravkin and Bogatov 1987; Starobogatov et al. 2004). Curiously, and although no type locality for this species was given, *Cristaria tuberculata* is the type species of the genus and usually listed as a junior synonym of *Cristaria plicata* (Leach, 1815) (Simpson 1914; Haas 1969).

*Cristaria herculea* (Middendorff, 1847) known from the Transbaikalia, in Far East Russia, occurs mainly in rivers and reservoirs with slow or no currents, in a variety of substrates, including gravel, sand and mud, being tolerant of silty conditions. The fish-hosts, necessary for glochidia metamorphosis, are still unknown. The conservation status of *Cristaria herculea* from the rivers of Transbaikalia was considered to be relatively stable during the last century. However, our research over the last ten years showed that the species has become very rare due to pollution and other anthropogenic impacts on rivers and habitats. Under this view, *Cristaria herculea* was included in the Red Book of Transbaikalsky territory (Klishko 2012).

To solve taxonomic issues when conchological characters (especially shell convexity) form the basis of species separation, it is relevant to test these uncertainties with molecular DNA sequence analyses. Under these assumptions, the aims of this paper were to study the morphological and anatomical characteristics of *Cristaria herculea* from the Upper Amur basin in Transbaikalia, to compare them with published *Cristaria* spp. ﻿data and to test the species level status of *Cristaria herculea* with the use of molecular data.

## Material and methods

### Sampling and morphometry

Specimens of *Cristaria herculea* were collected in 2008–2012 from the Shilka, the Nercha and the Onon rivers, from the Kharanorsky reservoir, situated in the upper reaches of the Amur (Transbaikalia, Russia), and also from Buir-Nur Lake (Mongolia) (Table 1). Foot tissue samples were collected from living mussels of the Onon River and Kharanorsky reservoir and were preserved in 96% non-denatured ethanol for molecular analyses. The following shell dimensions (mm) were measured in all collected animals: length, width, height at umbo and maximal height. For species identification, the ratio of maximal shell inflation to the distance from the umbo to the posterior end of the posterior tooth was determined, according the identification key by Zatravkin and Bogatov (1987) and Starobogatov et al. (2004).

**Table 1.** Shell morphometry characteristics of *Cristaria herculea* from the upper Amur River basin. L – shell length; H – maximal shell height; h – shell height measured from umbo; h_1_ – shell height measured from middle lateral tooth to ventral margin; l_1_ – the distance from umbo to posterior end of the lateral tooth; B – maximal shell inflation (width); R_1_ = B/l_1_, R_2_ = B/h_1_; n – number of measured shells; * – living mollusks dissected for the anatomical study and (n_1_) – their number.

Characteristics	Kharanorsky reservoir	Onon River	Nercha River	Shilka River	Buyr-Nur Lake
L, mm	128.7–290.0 258.0*	147.6–238.0 167.2*	120.2–174.0	152.0	148.9–151.0
H, mm	93.8–212.3 187.5*	94.9–124.8 102.0*	73.3–112.9	92.1	99.9–102.0
h, mm	65.3–148 130.6*	66.5–112.0 74.9*	51.6–80.0	66.3	69.2–71.1
h_1_, mm	75.0–161.2 152.5*	82.1–134.0 88.9*	68.7–98.1	82.3	63.1–64.9
l_1 _, mm	52.5–113.0 104.9*	58.0–99.4 66.9*	50.0–74.0	58.2	83.0–84.8
B, mm	41.2–88.0 79.4*	44.3–76.1 50.7*	39.0–53.9	47.1	46.0–48.0
B/L	0.316 ± 0.0055 0.308*	0.294 ± 0.0169 0.289*	0.317 ± 0.007	0.309	0.312 ± 0.0065
R_1_	0.760 ± 0.0311 0.756*	0.763 ± 0.0039 0.758*	0.768 ± 0.033	0.807	0.736 ± 0.0053
R_2_	0.540 ± 0.0119 0.521*	0.552 ± 0.0192 0.570*	0.561 ±0.0087	0.571	0.558 ± 0.0052
n (n_1_)	5 (1)	4 (1)	3	1	3

### DNA extraction, PCR and sequencing analyses

Whole genomic DNA was extracted from small tissue pieces of two individuals (preserved in 96% ethanol) using a standard high-salt protocol (Sambrook, Fritsch and Maniatis 1989). A fragment of ~700 bp of CO1 gene was amplified by PCR, using the primers LCO_22me2 and HCO_700dy2 (Walker et al. 2006, 2007) with PCR conditions described in Froufe et al. (2014). Amplified DNA temfigs were purified and sequenced by a commercial company, Macrogen, using the same primers. Chromatograms were checked by eye using ChromasPro 1.41 (technelysium.com.au) and the alignment was performed using Bioedit v5.0.9 (Hall 1999). For a preliminary analysis, all *Cristaria* sp. CO1 sequences available on GenBank were downloaded (n= 65). Afterwards, 52 of these sequences were excluded from the present analysis for clarity (they all represented different haplotypes that fell inside the *Cristaria plicata* clade, see results; data not shown). A final alignment was analysed, where the selected outgroups included one *Anodonta beringiana* individual and one *Sinanodonta woodiana* (Table 2).

**Table 2.** List of specimen samples sequenced (CO1) and GenBank accession numbers. *Unpublished

Species	Locality	Country	Code/GenBank	Study
*Cristaria herculea*	Onon River	Russia	Biv246	This study
*Cristaria herculea*	Charanorsky Reservoir	Russia	Biv247	This study
*Cristaria plicata*	Lower Yangtze	China	EU698893; EU698897; EU698913; EU698948	Jia and Li*
*Cristaria plicata*	Unknown	China	JF700152; JF700153	Zhang et al.*
*Cristaria plicata*	Zhejiang	China	FJ986302	Jiang, Zheng and Wang 2010
*Cristaria plicata*	Unknown	South Korea	GQ451860	Park et al.*
*Cristaria plicata*	Unknown	South Korea	GU944476	Lee et al. 2012
*Cristaria* sp.	Lower Yangtze	China	EU698909; EU698910; EU698940; EU698942	Jia and Li*
*Anodonta beringiana*	Jo-Jo Lake	Canada	DQ272370	Gustafson and Iwamoto 2005
*Sinanodonta woodiana*	Unknown	Poland	HQ283347	Soroka and Burzynski*

The final data set was then analysed using maximum likelihood (ML) and Bayesian inference (BI) methods. The best-fit model of nucleotide substitution evolution under corrected Akaike Information Criterion was estimated using JModelTest 2.1.4 (Darriba et al. 2012). Model GTR+I+G was chosen and used in the phylogenetic analyses. ML trees were built in RAxML 7.2.6 (Stamatakis 2006) running 1,000 bootstrap replicates and searching for the best-scoring ML tree. Phylogenetic BI was performed using MrBayes version 3.2.2 (Ronquist and Huelsenbeck 2003). Two independent runs 1 million generations long were sampled at intervals of 100 generations producing a total of 10,000 trees. Burnin was determined upon convergence of log likelihood and parameter estimation values using Tracer 1.6 (Rambaut and Drummond 2007). Estimates of sequence divergence (uncorrected *p*-distances) were assessed using MEGA 6 software (Tamura et al. 2013).

## Results

### Morphometry and species identification

The morphometric characteristics of *Cristaria herculea* are summarized in Table 1. The shell length of the collected *Cristaria* individuals ranged from 120 to 290 mm, the maximum shell height from 73 to 212 mm and the shell width was 39–88 mm. The ratio of maximal shell inflation to the distance, measured from umbo to posterior end of the lateral tooth (R_1_) was 0.76–0.81, and to shell height measured from the middle of the lateral tooth to ventral margin (R_2_) – 0.52–0.57. This ratio enabled the identification of the collected mussels as *Cristaria herculea*, according to the published keys (Zatravkin and Bogatov 1987; Starobogatov et al. 2004)

### Shell morphology

**Figure 1.**
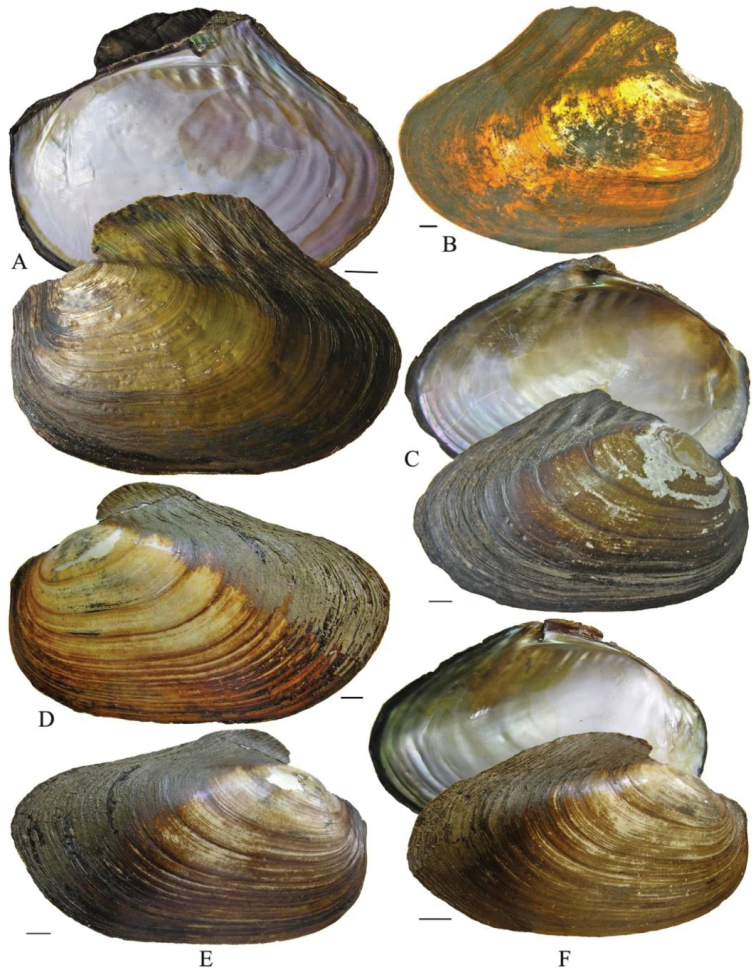
*Cristaria herculea* from Upper Amur River basin. **A, B** from Kharanorsky reservoir **C** from Buir-Nur Lake (Mongolia) **D** from Onon River **E** from Shilka River **F** from Nercha River. Scale bar 1 cm.

**Figure 2.**
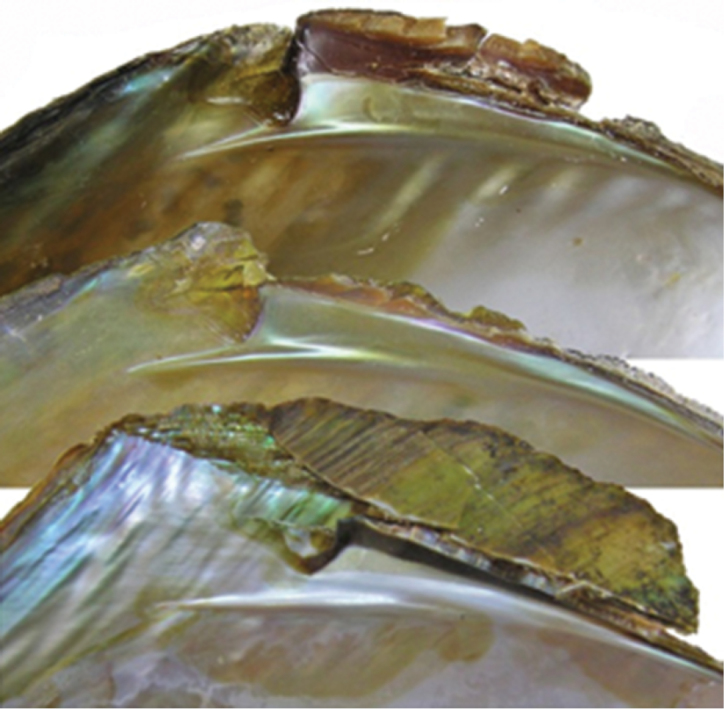
Lateral tooth of riverine shells (upper two) and from reservoir (lower).

**Figure 3.**
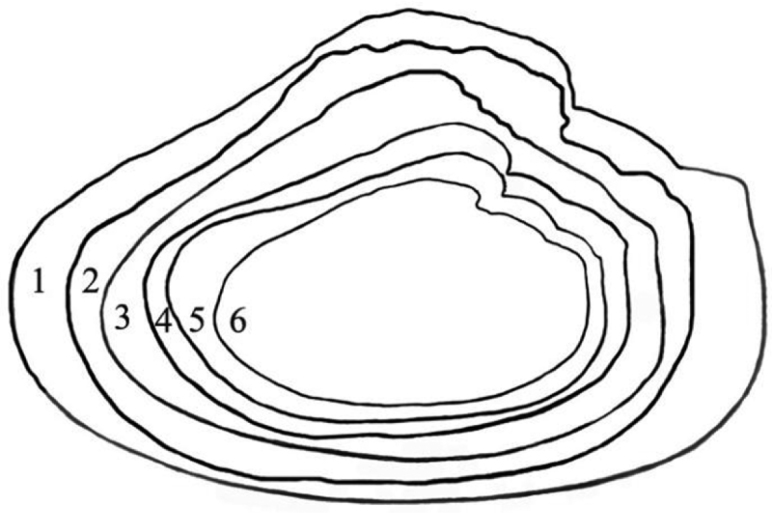
Shell outlines of *Cristaria herculea*. **1–3** (reservoir/lakes) **4–6** (rivers).

The shells from the reservoir are elongated diamond-shaped with a green-brown or red-brown coloured periostracum, with developed posterior dorsal wing and hardly expressed anterior wing (Fig. 1A, B). On the other hand, the shells from Buir-Nur Lake are oval-triangular with a dark brown or black coloured periostracum and with less developed or broken dorsal wing (Fig. 1C). The shells from the different river biotopes are elongated, oval-triangular, which may become oval when a dorsal wing is eroded or broken, alate, and with a periostracum colour that varies from yellow-brown to dark brown or black; the posterior dorsal wing is underdeveloped and the anterior dorsal wing is absent (Fig. 1D–F). Umbos are broad and slightly elevated above the dorsal margin. The umbo sculpture is presented in the form of a few sub-concentric bars. The dorsal margin behind the umbo turns into the high posterior wing, which is sometimes eroded or broken. There are large undulating folds or ridges on the posterior slope extending onto the posterior dorsal wing that are expressed more clearly in smaller specimens. The shell anterior margin is straight and the ventral margin may vary from slightly convex to straight or even slightly concave. The posterior margin in river shells is evenly rounded, slightly curved or concave when meeting the dorsal margin. The lateral teeth are straight or slightly curved, one in each valve (Fig. 2). While the anterior adductor scar is deep, the posterior is shallow and slightly visible. The nacre is blue, pale-pink, or yellow-pink with large olive spots. The shell outlines of river shells presented, in general, similar anterior margins with those from reservoirs but differ in having a smaller slope of the dorsal margin as seen in Fig. 3 (1–6).

### Soft body anatomy

**Figure 4.**
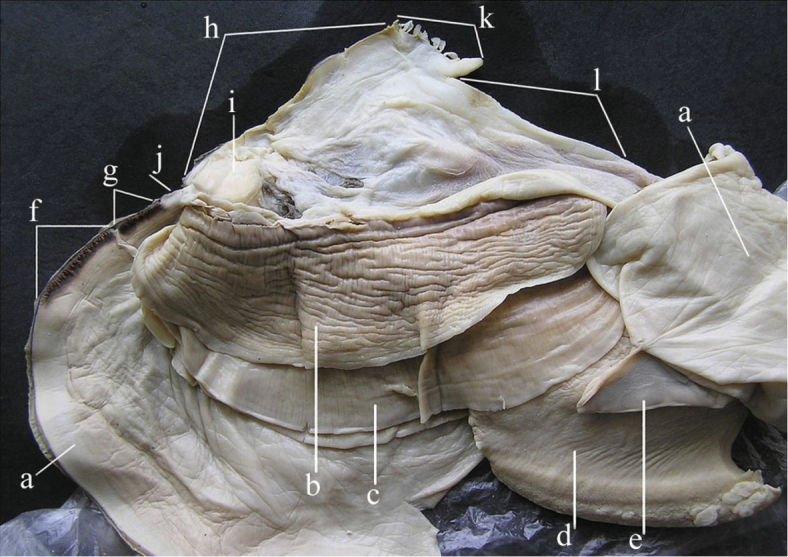
Morphology of *Cristaria herculea* soft body: **a** – mantle, **b** – outer gill, **c** – inner gill, **d** – foot, **e** – labial palps, **f** – incurrent aperture, **g** – excurrent aperture, **h** – supra-anal aperture, **i** – dorsal adductor muscle, **j** – mantle bridge, **k** – dorsal wing mantle projections, **l** – muscular anterior margin of dorsal mantle wing.

**Figure 5.**
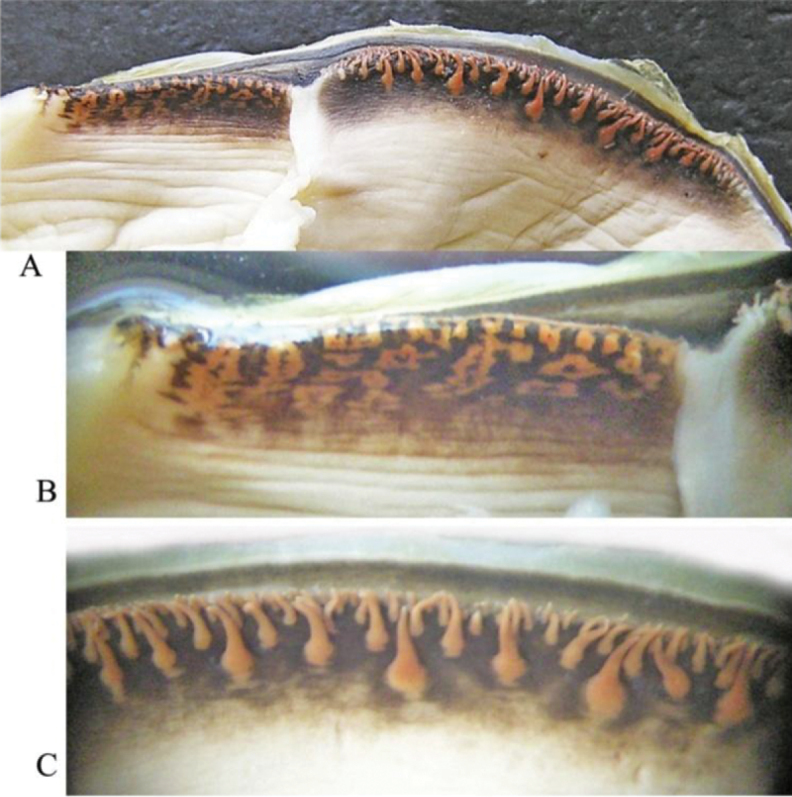
*Cristaria herculea*: **A** excurrent aperture (to the left) and incurrent aperture (to the right) **B** excurrent aperture (magnification) **C** shape of papillae in incurrent aperture (magnification).

**Figure 6.**
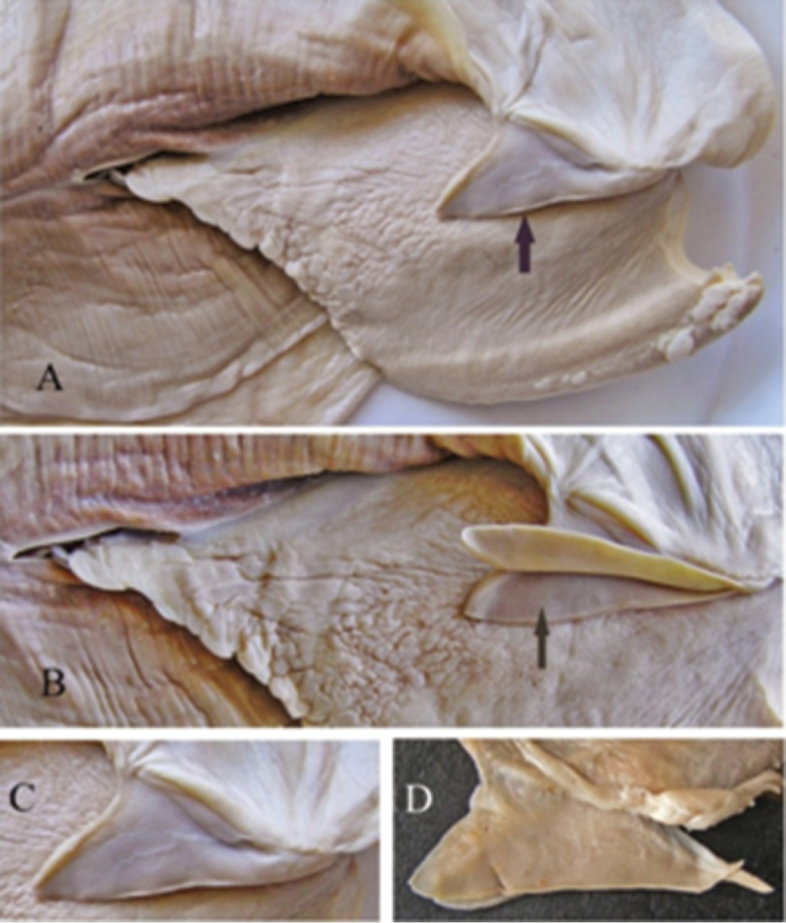
Labial palps of *Cristaria herculea* (**A, B, C**) and of *Sinanodonta* sp. (**D**).

**Figure 7.**
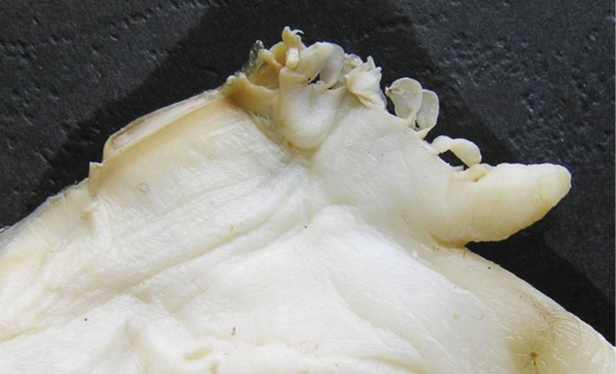
Dorsal mantle wing of *Cristaria herculea* with comb-shaped projections.

A general view of the whole soft body morphology of *Cristaria herculea* is shown in Fig. 4. Mantle colour is creamy white, with black or brown edges (Fig. 4a). Dorsal mantle margin presents a well expressed high angle with a comb-shaped projections on the top (Fig. 4k) and a muscular anterior margin (Fig. 4l). Gills are creamy white or light brown; dorsal margin is straight to sinuous and ventral margin is moderately convex. Inner gills are much longer and higher than outer gills (Fig. 4b, c); gill length is 46–54% of shell length, gill height is 25–40% of gill length and outer gill height is 67–75% of the inner gill height. The foot is massive, creamy white and darker distally (Fig. 4d). Labial palps are triangular, creamy white to blue-grey, straight or slightly convex dorsally; straight or gently concave ventrally and bluntly pointed ventrally (Fig. 4e). Labial palp length is 3.9–4.2% of inner gill length and labial palp height 34–35% of labial palp length. Incurrent aperture (Fig. 4f) is longer than the excurrent aperture (Fig. 4g) and shorter than the supra-anal aperture (Fig. 4h). Supra-anal aperture opening is located from the dorsal margin of the posterior adductor muscle (Fig. 4i) to the posterior dorsal edge of the posterior mantle wing. Supra-anal aperture length is 20–25% of the shell length or double the length of the incurrent aperture; it is creamy white to pearly white inside, with a very thin yellow-brown marginal band. Mantle bridge (Fig. 4j) separates the excurrent from the supra-anal aperture and is 8–10% of the supra-anal aperture length. Incurrent aperture length is 11–13% of the shell length, is creamy white to light tan within, with a combination of orange, brown and black basal to the papillae and to the bands margin which may present a reticular pattern. Excurrent aperture length is 46% of incurrent aperture length, colour is creamy white within with black or dark brown edges basally, margin papillate; have irregular mottled pigmentation of some combination of dark brown and orange (Fig. 5A–C). Papillae of the incurrent aperture are located in 3–4 rows, linear-fusiform in shape, mostly simple, with thickening of the papilla basement in the first and second medial rows, dark-orange; papillae of outer or lateral rows are shorter and more numerous (Fig. 5C). Labial palps of *Cristaria herculea* and *Sinanodonta* sp. are morphologically distinct (Fig. 6A–D). The anterior acuminate edges of *Sinanodonta* sp. labial palps are not completely attached to the mantle (Fig. 6D) in contrast with those on *Cristaria herculea* (Fig. 6A–C). The distinctive feature of the genus *Cristaria* within the tribe Anodontini is the posterior dorsal mantle wing and projections (Fig. 7). The comb-shaped projections are dorsal extensions of the mantle that penetrate into the cavities of shell wing, to provide for the wing growth.

### Phylogenetic analysis

**Figure 8.**
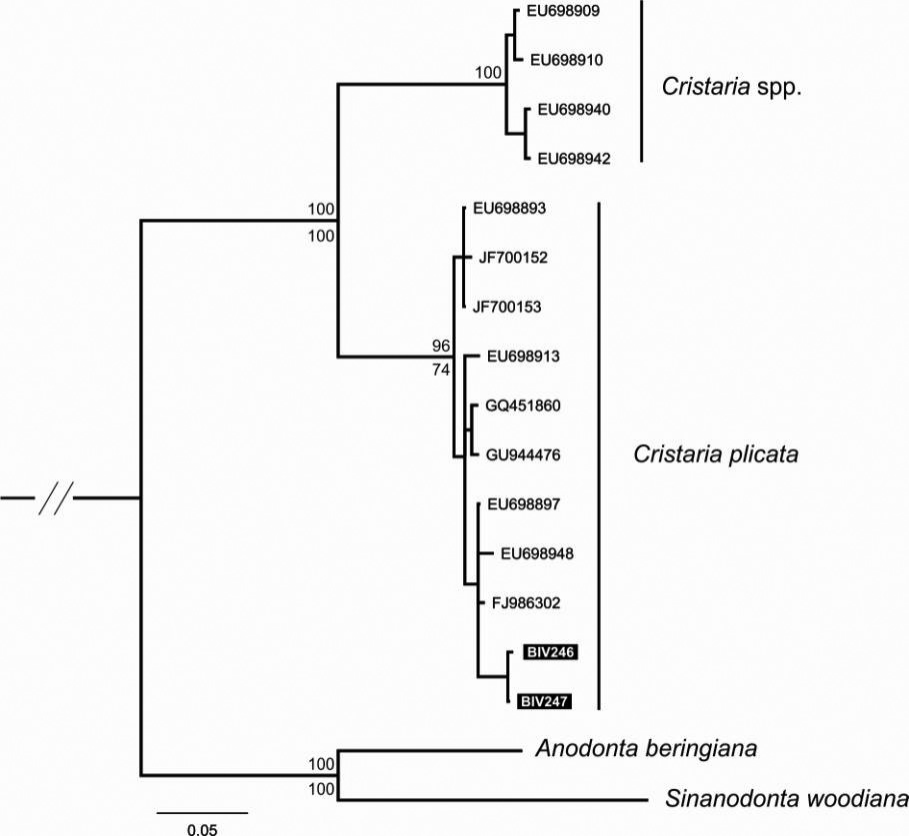
Phylogenetic tree obtained by Bayesian Inference and Maximum Likelihood analyses, using mtDNA fragments (CO1). Support values are given as Bayesian posterior probability above nodes and as bootstrap support below nodes, except for those within major clades, which have been omitted for clarity. Available sequences downloaded from GenBank and new sequences codes refer to Table 2.

Aligned CO1 sequences had a total length of 620 bp, with 143 polymorphic and 92 parsimony informative sites. No indels and no unexpected stop codons were observed after translating all sequences to amino acids. The tree topologies resulting from the single tree recovered from ML and BI approaches were congruent, and results of both analyses are shown in Figure 8. Two major mtDNA clades were retrieved with strong support: one includes all the individuals from *Cristaria plicata*, including the new sequences collected for this work (Biv246 and Biv247; Fig. 8) and the other includes six individuals also originally assigned to *Cristaria plicata* (Jia and Li, Unpublished). However, it is obvious that the phylogeny of the *Cristaria* genus needs further evaluation, since these individuals are 8.9% (uncorrected *p*-distance) from the others, strongly indicating the existence of two different *Cristaria* species in this data set. Thus, this clade is here referred as *Cristaria* sp.

## Discussion

The whole shell morphology of *Cristaria herculea* from the Upper Amur basin described here is very similar and corresponds to that previously described by Zhadin (1938 1965) for *Cristaria plicata*. Relative to *Cristaria tuberculata*, Prozorova and Sayenko (2001) contend that, although shell shape differences are considerably smoothed with age or in more dynamic habitats, there are enough conchological differences and divergence in ecological preferences to distinguish them easily using both morphological and ecological characters. On the other hand, while many early researchers gave special importance to soft body anatomical characters in freshwater bivalves for taxonomic research (e.g. Ortmann 1923; Reardon 1929; Fuller and Bereza 1975; Kat 1983; Bogan 1992), there is no published information about anatomical differences between these two species. The soft body anatomy described here for the *Cristaria herculea* from Transbaikalia and the previously described *Cristaria herculea* from the Far East (Khanka Lake) (Sayenko and Bogatov 2004) are similar, although the papillae of the incurrent aperture of *Cristaria herculea* from Khanka Lake (Far East) have no thickening and are located more closely in contrast to Transbaikalian ones (Sayenko and Bogatov 2004) (Fig. 5A–C).

The study of *Cristaria herculea* and *Cristaria tuberculata* glochidia has also shown no differences in shell size and proportions or in the disposition of macro spines on the distal end of hooks (Sayenko 2006). Sayenko noted that the morphological indices of *Cristaria herculea* and *Cristaria tuberculata* glochidia, when compared with the indices of *Cristaria plicata* from China and similar species from Japan, are within the same size and shape range limits. Under this view, the selected morphological features of glochidia, on which is based the separation of species *Cristaria herculea*, *Cristaria tuberculata*, and *Cristaria plicata* are varied and probably cannot be considered as systematic. Furthermore, no differences were revealed in the reproductive cycle timing of *Cristaria* species from Khanka Lake (Far East Russia) and Biwa Lake (Japan). In both lakes, reproduction may last from October to April (Higashi and Hayashi 1964; Chernyshev 1996; Prozorova and Sayenko 2001). In another study, based on the similarity level analyses of the electrophoretic patterns of myogens, ﻿Kodolova and Logvinenko (1988) concluded that both *Cristaria tuberculata* and *Cristaria herculea* belong to one single species. More recent publications by Graf (2007) and He and Zhuang (2013) based on morphological observations, also state that *Cristaria tuberculata* and *Cristaria herculea* represent a single species, *Cristaria plicata*. The synonymy of *Cristaria herculea* from the Transbaikalia with *Cristaria plicata* is confirmed in the present study since they both fall inside the diverse *Cristaria plicata* clade. An additional clade was retrieved with sequences from the Yangtze basin (China) mussels. Although these animals were originally described as *Cristaria plicata*,﻿ they belong to a distinct *Cristaria* sp. which may refer to either *Cristaria tenuis* (Griffith & Pidgeon, 1833) or *Cristaria radiata* Simpson, 1900, both present in the Yangtze River basin (He and Zhuang 2013). Furthermore, the newly sequenced *Cristaria herculea* individuals collected from Transbaikalia are five mutations away from the closest Yangtze haplotype (data not shown). Thus, more individuals from the Transbaikalia, including the vast Amur basin, as well as specimens from Far East Asia (e.g. Khanka Lake) are needed to determine if they form a distinct evolutionary unit (e.g. subspecies) within *Cristaria plicata*.

In summary, the shell morphology, anatomy, and known ecological traits of *Cristaria herculea* from the Upper Amur basin are similar to those described for *Cristaria plicata*. Additionally, the CO1 molecular analysis confirms *Cristaria herculea* as a synonym of *Cristaria plicata*. As for *Cristaria tuberculata*, while some studies reported similar morphological and molecular characters to *Cristaria herculea*, other authors reported differences not only in shell shape (inflation) but also in ecological requirements and morphological features of the glochidia, suggesting the occurrence of two distinct species in Far East Russia. Therefore, the distinction of *Cristaria tuberculata* from *Cristaria herculea*/*Cristaria plicata* is still an open question that should be carefully investigated using additional molecular data.
